# Perceived Conventionality in Co-speech Gestures Involves the Fronto-Temporal Language Network

**DOI:** 10.3389/fnhum.2017.00573

**Published:** 2017-11-30

**Authors:** Dhana Wolf, Linn-Marlen Rekittke, Irene Mittelberg, Martin Klasen, Klaus Mathiak

**Affiliations:** ^1^Department of Psychiatry, Psychotherapy and Psychosomatics, Medical Faculty, RWTH Aachen, Aachen, Germany; ^2^Natural Media Lab, Human Technology Centre, RWTH Aachen, Aachen, Germany; ^3^Center for Sign Language and Gesture (SignGes), RWTH Aachen, Aachen, Germany; ^4^JARA-Translational Brain Medicine, RWTH Aachen, Aachen, Germany

**Keywords:** fMRI, inferior frontal gyrus, inter-subject covariance, naturalistic stimuli, semiotics

## Abstract

Face-to-face communication is multimodal; it encompasses spoken words, facial expressions, gaze, and co-speech gestures. In contrast to linguistic symbols (e.g., spoken words or signs in sign language) relying on mostly explicit conventions, gestures vary in their degree of conventionality. Bodily signs may have a general accepted or conventionalized meaning (e.g., a head shake) or less so (e.g., self-grooming). We hypothesized that subjective perception of conventionality in co-speech gestures relies on the classical language network, i.e., the left hemispheric inferior frontal gyrus (IFG, Broca's area) and the posterior superior temporal gyrus (pSTG, Wernicke's area) and studied 36 subjects watching video-recorded story retellings during a behavioral and an functional magnetic resonance imaging (fMRI) experiment. It is well documented that neural correlates of such naturalistic videos emerge as intersubject covariance (ISC) in fMRI even without involving a stimulus (model-free analysis). The subjects attended either to perceived conventionality or to a control condition (any hand movements or gesture-speech relations). Such tasks modulate ISC in contributing neural structures and thus we studied ISC changes to task demands in language networks. Indeed, the conventionality task significantly increased covariance of the button press time series and neuronal synchronization in the left IFG over the comparison with other tasks. In the left IFG, synchronous activity was observed during the conventionality task only. In contrast, the left pSTG exhibited correlated activation patterns during all conditions with an increase in the conventionality task at the trend level only. Conceivably, the left IFG can be considered a core region for the processing of perceived conventionality in co-speech gestures similar to spoken language. In general, the interpretation of conventionalized signs may rely on neural mechanisms that engage during language comprehension.

## Introduction

Languages are generally seen as conventional sign systems par excellence. Linguistic signs are primarily spoken and written words, or manual signs in the case of sign language. However, face-to-face communication typically not only includes symbolic linguistic signs, but also facial expressions, eye gaze and co-speech gestures, all of which contribute information to the overall message (e.g., Kendon, 1994). Some gestures, like emblems, express a conventional meaning without depending on the concurrent speech (e.g., McNeill, 1992). By contrast, most co-speech gestures are not self-explanatory but need to be interpreted in relation to the concurrent speech and other situational context (e.g., Cienki and Müller, 2008; So et al., 2009; Calbris, 2011). Nonetheless, some co-speech gestures exhibit varying degrees of conventionality in that they convey aspects of the objects, actions, or abstract relations in similar manners (movement pattern or shape; Peirce, 1960; Mittelberg, 2006). There is a growing body of neuroimaging research on sign language and emblems on the one hand and on co-speech gestures on the other hand (for review, see e.g., Yang et al., 2015). However, the neural mechanisms underlying the perception of conventionality in co-speech gestures remains largely unknown.

### Co-speech gestures and the language networks

Co-speech gestures vary in their semantic functions and in their degree of conventionality. McNeill introduced a typology of co-speech gestures that distinguishes between iconic, metaphoric, deictic, cohesive, and beat gestures (McNeill, 1992, 2005). The perception of these types of co-speech gestures have been studied within narratives (e.g., Skipper et al., 2007; Wilson et al., 2008; Dick et al., 2009) or in isolation (e.g., Hubbard et al., 2009; Straube et al., 2010; Dick et al., 2014). However, detailed accounts are available mainly on iconic gestures, which portray concrete entities or actions (for a review, see Özyürek, 2014); they yield recruitment of inferior frontal, superior and middle temporal gyri, posterior temporal cortex, as well as motor cortex. Based on McNeill's typology, experimental studies have identified distinct neural correlates for location-related gestures as compared to form-related gestures (pointing and iconic; Nagels et al., 2013) as well as for abstract vs. concrete meaning (metaphoric and iconic; e.g., Straube et al., 2013). Gestures of each type may comprise conventional aspects (Bressem and Müller, 2014; Ladewig, 2014), but so far this has not been the focus of empirical investigations.

Co-speech gestures are processed within the inferior frontal and superior temporal cortex (IFG and pMTG/STG; Bernardis and Gentilucci, 2006; Dick et al., 2009; Straube et al., 2012), reflecting the major functional nodes of the language network described by Broca and Wernicke. The IFG and pMTG/STG have frequently been reported in studies investigating the neural correlates of co-speech gestures; both during actor-performed story telling [usually iconic, metaphoric, deictic, beats, and self-adaptors (e.g., Wilson et al., 2008; Dick et al., 2009, 2014) and in response to specific, isolated gesture types: iconic (Willems et al., 2009; Straube et al., 2012), metaphoric (e.g., Kircher et al., 2009; Nagels et al., 2013), deictic (e.g., Nagels et al., 2013), and beat gestures (e.g., Hubbard et al., 2009; Biau et al., 2016)]. An ALE meta-analysis of functional imaging studies on hand gesture comprehension implicated the IFG and pMTG/STG in the conceptual processing of semantic components of co-speech gestures (Yang et al., 2015). In fact, the conventional emblems particularly recruit the IFG and superior temporal cortex (e.g., MacSweeney et al., 2002; Xu et al., 2009; Andric et al., 2013). Further, in participants naïve to sign language, left IFG and pSTG responded to videos of spoken and signed (French Sign Language) narratives alike, rendering them candidates for a cross-modal processing hub of conventional concepts (Courtin et al., 2011). Taking a semiotic perspective, conventionality may be one of the common semiotic dominators for understanding semantic aspects of co-speech gestures.

### Co-speech gestures and conventionality

A gesture acts as a communicative sign and can thus be described using sign theory (semiotics). On a cognitive-semiotic level, conventionality may be accounted for by drawing on Peirce's model of Universal Categories (UCs; Peirce, 1955, 1960). These semiotic categories describe cognitive categories (Holenstein, 2008); thus they are amenable to cognitive-behavioral and neuroscientific testing (Paolucci, 2011; Galantucci et al., 2012; Zlatev, 2012). Peirce's theory has been considered one of the most appropriate semiotic framework for the study of cognitive processes (Daddesio, 1994; Stjernfelt, 2007; Fusaroli and Paolucci, 2011; Sonesson, 2014). This pragmaticist approach to communication by signs is not solely derived from language and may thus account for both speech and visuo-spatial modalities such as actions and visual images (Jensen, 1995; Nöth, 2001). It is particularly well suited for neuroimaging studies of communication because it emphasizes the perspective of the interpreting mind (Peirce, 1955), represented by the observers' brain activity. Peirce's UCs have inspired theoretical accounts developed to describe and interpret manual gestures (e.g., McNeill, 2005; Fricke, 2007; Mittelberg, 2013a; Mittelberg and Waugh, 2014), and also have motivated observational studies in the field of multimodal communication. The sign-object relation is the best known aspect of the UCs, and informed typologies for empirical analyses of manual gestures (e.g., as “iconic,” “deictic,” or “emblematic” gestures; McNeill, 1992) which have been amply used in behavioral and neuroimaging studies on gesture perception and comprehension (for reviews, see Özyürek, 2014; Wagner et al., 2014; Yang et al., 2015).

The current study addresses perceived conventionality as afforded by Peirce's UC “Thirdness” during the interpretation process. The Thirdness category (see Discussion section for Firstness and Secondness) pertains to rules, laws, patterns, and habits such as underpinning conventional meanings of spoken words and emblematic gestures, but also social norms, rituals, etc. (Peirce, 1960; Potter, 1967; Mittelberg, 2006). For the present understanding of conventionality, habits and embodied structures of experience play a central role, such as action routines and other patterned way of interacting with the environment. These patterns may be based on movements and the handling of objects. While gesture interpretation depends on the context such as the interaction with speech and other communicative signs, we expect, in accordance with the semiotic model applied here, similar response patterns within a culturally homogeneous group of language users (see Bressem and Müller, 2014; Ladewig, 2014 on recurrent gestures in German). The present study aimed to modulate the response amplitudes of the neural correlates. Therefore, a task directed the participant's focus to the processing of conventionality in co-speech gestures. Importantly, utilizing a task enabled an analysis independent of expert-rated gesture types.

### Instructional tasks and conventionality in co-speech gestures

Tasks may not only induce different levels of attention or distraction (e.g., Mathiak et al., 2005) but they may lead to changes in neural activation as well (Mathiak et al., 2007). They modulate functional responses to basic sensory processing (Chawla et al., 1999; Mathiak et al., 2004) and also to more complex cognitive processes such as mental imagery, perspective taking, and semantic processes (Cooper et al., 2011; Lindenberg et al., 2012; Lahnakoski et al., 2014). The effectiveness of tasks in directing the perception of signs and specific stimulus aspects is well documented for experiments with a free viewing paradigm and complex, naturalistic stimulus material (Cooper et al., 2011; Lahnakoski et al., 2014). For instance, brain activation during comprehension of identical auditory narratives was affected by tasks that guided the participant's attention to either space-, time-, or action-related information (Cooper et al., 2011). When presented with the more complex naturalistic stimuli such as film clips inter-subject synchronization has been modulated by perspective taking (Lahnakoski et al., 2014). Inter-subject correlation (ISC) predicted the participant's perspective of either a detective or an interior decorator while watching short video clips. In higher order visual processing areas (lateral occipital cortex, ventral temporal cortex) and posterior parietal cortex, neural activation patterns were more similar in participants with the same perspective. Instructional tasks modulated the neural systems processing emblematic gestures as well. Taking the perspective of the sender or the receiver the altered activation in the inferior and medial frontal cortex (Lindenberg et al., 2012) and classifying an emblem according to meaningfulness vs. simple categorization impacted a right-hemispheric network including inferior parietal, inferior temporal and superior temporal cortex (Nakamura et al., 2004). Thus, the interpretation of verbal and gestural information can be modulated by task instructions.

Most studies on neural processing of co-speech gesture perception gave unspecific instructions such as “to watch” (e.g., Wilson et al., 2008; Straube et al., 2010; Dick et al., 2014), or a color discrimination task unrelated to the gestures (e.g., Straube et al., 2014). Only one study asked participants to decide between dominant or subordinate meaning of verbal homonyms on the basis of the accompanying, disambiguating gesture (Holle et al., 2008). This linguistic task increased activation in the left pSTS, bilateral inferior parietal lobule and bilateral ventral precentral sulcus. However, also this study did not compare between tasks. Thus, no direct evidence was available for the top-down modulation of the processing of co-speech gestures.

In the current study, novel tasks modulate the processing of co-speech gestures presented in video clips of spontaneous story retellings. To account for the complexity of naturalistic communication, we investigated functional covariance across times series and assessed task effects on the resulting ISC maps.

### Gestures and ISC mapping

As concerns the naturalistic production of co-speech gestures, studies into its neural processing warrant multimodal stimuli such as video clips. For these stimuli, a general linear model represents only aspects of complex and interactive stimuli (Mathiak and Weber, 2006). Successful alternatives are methods that do not need explicit temporal models such as independent component analysis (Zvyagintsev et al., 2016) and ISC (Hasson, 2004). Providing means for direct hypothesis testing, ISC is particularly well suited to capture synchronous changes in implicated networks and is well established for naturalistic stimuli (Hasson, 2004; Jääskeläinen et al., 2008; Bhavsar et al., 2014). One previous ISC study presented multimodal story retellings without variation of the task (Wilson et al., 2008). The multimodal narrations yielded significant ISC in superior temporal areas, cingulate cortex, medial and inferior frontal regions, precuneus and premotor regions. In contrast, the direct comparison of activity during stimulus presentation with baseline in a general linear model revealed activation in the superior temporal areas only. This finding further evidences the sensitivity and specificity of ISC to measure neural involvement during the free viewing of naturalistic stimuli.

### Hypotheses

The goal of this study was to investigate the neurocognitive underpinnings of perceiving conventionality in a broad range of naturally occurring co-speech gestures. To this end, study participants watched video clips of movie retellings, comprising spontaneously produced co-speech gestures in a behavioral and an fMRI experiment. Participants attended to conventionality in co-speech gestures or to a control aspect of the perceived gestures, i.e., to any hand movement or to a relation between gesture and utterance. In the behavioral part of the experiment, participants watched the narrations and gave a button-response whenever they detected a gesture matching the task. Further, after watching a pair of videos with the same task, they judged which of the film clips comprised more of the specific gesture type. During the fMRI part, participants attended to the same aspects of co-speech gestures but replied only to the comparison task. We tested two hypotheses:

(1) Detection of conventional aspects in co-speech gestures is consistent across study participants and distinct from attending to non-conventional gestures. Therefore, we expected increased synchronization of button-press time series and of functional brain activation during the perception of conventionality in co-speech gestures. This tests the reliability of the conventionality detection task.

(2) The task to detect conventional co-speech gestures increases neural synchronization in key nodes of the language networks, that is, the left IFG (Broca's area) and the left pSTG (Wernicke's area). Further, we explored whether other neural structures may be involved in the detection of conventional aspects in co-speech gestures.

## Methods

### Study participants

Thirty-six right-handed German native speakers participated in the present study (18 women; age 19–35 years, mean: 24.8 years, SD 3.9). Participants had normal or corrected to normal vision, normal hearing, no history of psychiatric or neurological illness, no current psychopharmacological treatment, and no contraindication against MR investigations. The experiments were designed according to the Code of Ethics of the World Medical Association (Declaration of Helsinki, 2008) and the study protocol was approved by the local Ethics Committee. After detailed briefing and instruction, all participants gave written informed consent.

### Stimuli

**Figure 1:**
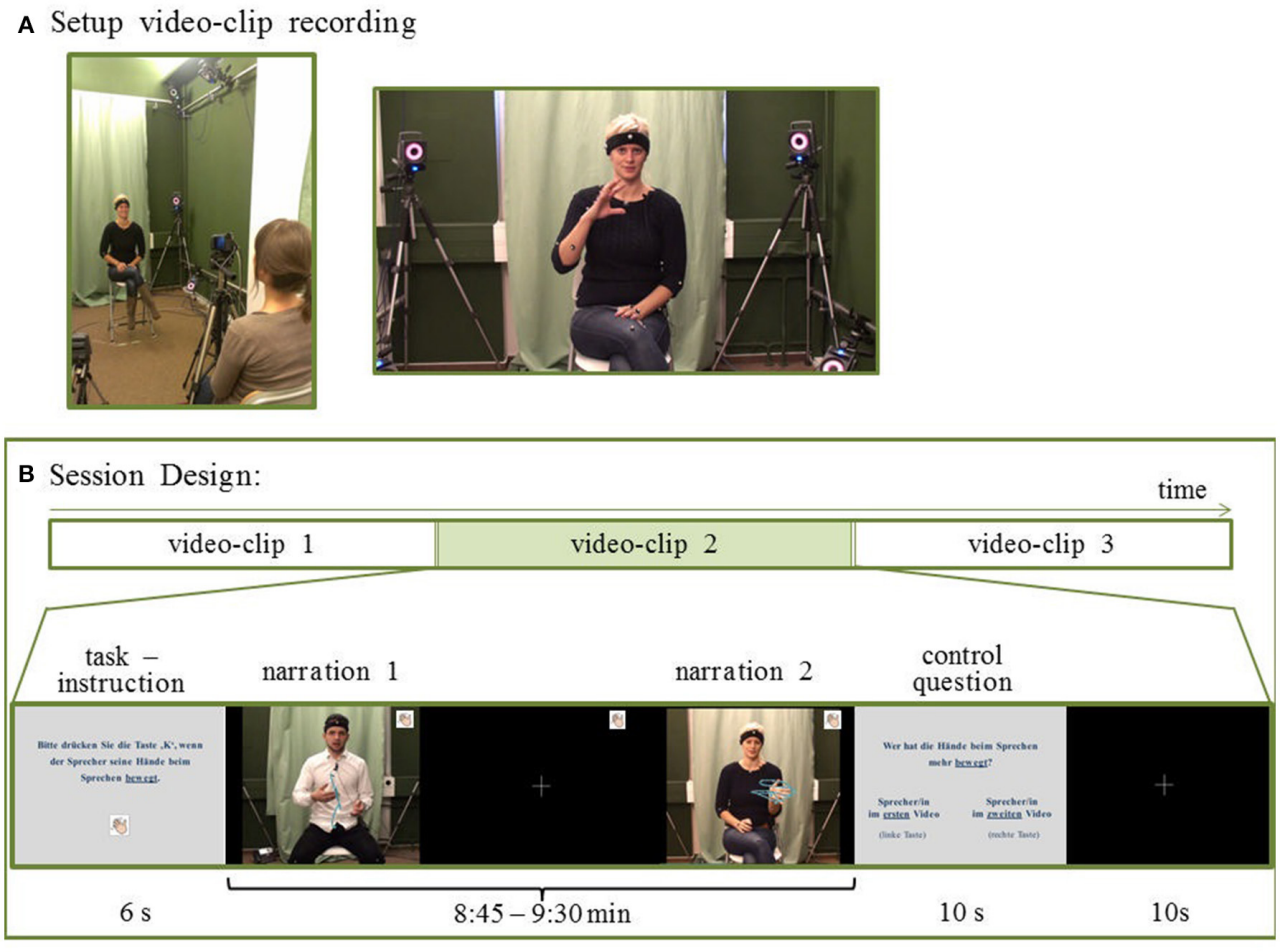
Stimuli for behavioral and fMRI experiment. **(A)** After watching short movies, non-professional speakers retold the narratives on video. 24 stories were combined into 12 video clips (9 min each). **(B)** Session design: Participants underwent 2 sessions per experiment. In one session, 3 video clips were presented. Each video clip was preceded by a task description and followed by a corresponding control question. For the behavioral part participants pressed a button according to the task. During the fMRI part they attended to the task (without pressing a button). For the CON task participants attended to conventional aspects in co-speech gestures, such as the expression of the relevance of a topic (compare “pragmatic gestures,” e.g., Payrati and Teßendorf, 2014) as indicated in the left still frame, or a depiction of a stepwise process (compare “image schemas in gestures,” e.g., Mittelberg, 2013a) as displayed in the right still frame. For visualization purposes the complete hand movement is indicated here by motion capture trajectories (blue lines). The trajectories were not displayed during the experiments. Written consent was obtained from the participants for publication of this image.

Video clips of short narrations, comprising spontaneously produced co-speech gestures, served as stimuli. Therefore, 14 right-handed German native speakers (6 women) were video-recorded during freely retelling the narrative of 3 short movies (“Lebensmotiv Tanz,” Balmas, 2013; “Der Archivar,” Cherdchupan, 2012; “What happened in Room 13,” Dila, 2007) directly after watching them (Rekittke et al., 2015). The order of the short movies was balanced across narrators. The narrator was seated in front of a video camera and a listener sat directly behind the camera with the camera at eye level (Figure 1A). The listener was instructed to follow the narration, but not to intervene while listening. For each short movie, the narrators were instructed to freely retell the narrative in a monolog immediately after watching. The narrator's speech and gesturing were not restricted or regulated by instructions, such that they could display their usual conversation behavior.

From the 42 video recordings, 24 narrations were selected as stimulus material. For the selection, we considered the recording quality, the duration of the narrative, as well as the gender balance of the narrators. Durations of selected narrations ranged between 1:58 and 7:24 min. These narrations were paired to yield 12 video clips that were about 9 min long (9:16 min ± 11 s, range 8:45–9:30 min). The 2 narrations in each video clip were separated by a 4-s fixation-cross phase.

Although we analyzed the fMRI data with model-free ISC, gesture units, phrases, and phases were annotated (Kita et al., 1998; Kendon, 2004) for a quantitative assessment. In total, 856 gesture units (71.3 ± 25.7, mean ± SD per block) with 1,877 gesture phrases (156.4 ± 36.3) occurred in the 12 video recordings. 1,830 (152.5 ± 36.3) phrases were annotated as strokes. In general, the videos comprised all of McNeills proposed gesture types (emblem, beat, deictic, iconic, and metaphoric gestures; McNeill, 1992, 2005). As it is common in freely produced co-speech gestures, emblems could be observed only rarely.

### Experimental design and procedure

Upon arrival, participants were instructed about the experimental procedure. Each participant took part in the behavioral experiment and subsequently in the fMRI experiment. During both parts, 6 video clips were presented in 2 sessions of 3 video clips each. After the experiment participants were debriefed and received their financial recompensation. The joint behavioral and fMRI experiment, with a break in between, lasted 3 h in total.

Since a button-press response is a finger tapping movement, it may cause motor-related artifacts in cortex areas relevant for gesture processing (e.g., premotor cortex, inferior frontal cortex). Therefore, we separated the behavioral data acquisition (behavioral experiment) from the neuroimaging data acquisition (fMRI experiment). The 12 video clips were divided into 2 sets of 6 videos clips each. Half of the participants (Group 1, 18 participants) watched the first 6 video clips during the behavioral part and the other 6 during the fMRI part; in the other half of the participants (Group 2, 18 participants), this relation was switched. This way, both behavioral and fMRI data was obtained for all video clips and participants watched every narration only once. Across the experiments each of the 12 video clips was matched to 2 of the 3 tasks (see section Task Instructions for details on the tasks), yielding 24 video clip—task combinations. Each task was thereby assigned to 8 video clips. By matching 2 tasks to 1 video clip we were able to perform an across-task comparison while keeping the stimuli identical. Differences in functional covariance could thus be ascribed to task effects rather than video clip effects. Matching only 2 of the three tasks to a video clip instead of performing complete permutation reduced the number of video clip-task combinations and, therefore, increased the number of participants watching the same combination yielding increased power. Effects of different videos were fully accounted for because covariance calculation compared only data from the same video clip. In total, 1 video clip—task combination was seen by 9 participants. To compensate sequence effects, the order of the video clip—task combinations was balanced across experiments.

Before a video clip, task instructions were given for 6 s. After the clip, a question was presented (10 s) inquiring in which of the 2 narration the participant discerned more of the task-relevant gestures. Responses were given via a button press on a keyboard during the behavioral experiment and on an MRI-compatible keyboard (Lumitouch) during fMRI. Each question was followed by a 10-s fixation cross (see Figure 1B for a schematic overview of 1 session).

### Task instructions

For each video clip the participants received 1 of 3 tasks to detect specific aspects of the presented narrations (Table 1 specifies the tasks). During the behavioral experiment, participants were instructed to pay attention to the video clips and press a button whenever they detected a gesture matching the given task. This button press marked the time point when a gesture was perceived as matching the task. During fMRI, the participants were asked only to attend to any gesture that matches the observation task but to refrain from pressing a button. The conventionality task was to detect conventional aspects of gestures (task CON).

**Table 1:** Task instructions.

**Task**	**Gesture-related attributes**	**Wording**
		“Please press the button if the speaker…” [Ger.: “Bitte drücke den Knopf wenn der Sprecher…”]
Conventionality [CON]	Conventional aspects in gesture	“…produces a hand movement which is commonly used” [Ger.: “…eine Handbewegung macht, die allgemein gebräuchlich ist.”]
Control 1 [ANY]	Physical event of hand movement	“… moves the hands during speaking.” [Ger.: “…seine Hände beim Sprechen bewegt. ”]
Control 2 [REL]	Disambiguation of gesture by accompanying speech	“…produces a hand movement which has a connection to the speech.” [Ger.:“…eine Handbewegung macht, die eine Verbindung zum Gesprochenen hat.”]

Participants could identify any hand movement they perceived as carrying conventional aspects. In order to insure naïve perception, we did not educate them about expert ideas on conventional gestures (e.g., the definition of emblems), expert definition of gesture types (such as based on McNeil's typology), or gesture morphology (e.g., form parameters, preparation, and stroke phases). During the behavioral experiment the wording was “Please press the button when the speaker produces a hand movement, which is in common use.” (Ger.:“Bitte drücke den Knopf, wenn der Sprecher eine Handbewegung macht, die allgemein gebräuchlich ist.“). This wording directly relates to Peirce's definition of Thirdness encompassing habits, patterns and rules in sign use. During the control conditions, participants focused on the occurrence of any hand movements (“…when the speaker moves the hands during speaking;” Ger.: “…wenn der Sprecher seine Hände beim Sprechen bewegt;” task ANY), and on gestures relating to the spoken content (“…the speaker produces a hand movement which has a relation to the speech;” Ger.: “…wenn der Sprecher eine Handbewegung macht, die eine Verbindung zum Gesprochenen hat;” task REL). With the first control task (ANY) we controlled for the processing of hand movements, while with the second control task (REL) we controlled for the audiovisual integration of gestures and concurrent speech.

During fMRI, the instruction was to only attend to the task-instructed aspects and not to press a button during watching. In both experiments, after each video clip the participants answered the control question by deciding in which of the 2 narrations they detected more of the indicated gestures. Task instructions were shown to the participants directly before the presentation of each video clip. Additionally, the tasks were explained in detail during a standardized briefing prior to the experiments. During this briefing, the instructor carefully refrained from moving her own hands and from giving gesture examples.

### Behavioral data acquisition and analyses

Participants were seated in front of a notebook with 15′ screen in a dimmed and quiet room. Sound was presented via headphones; the volume was adjusted to a comfortable hearing level. Presentation software® (Version 17.2, www.neurobs.com) was used to present the stimuli and to record button presses.

The continuously recorded button presses were analyzed as time series. The frequency of button presses were normalized between the task-conditions to an average of 17 per video by randomly omitting exceeding button presses. Thereby, we accounted for the variation in number of button press responses across the task conditions and simultaneously conserved the relative density. This procedure was only done for the behavioral experiment since no button press responses were recorded in the fMRI part. The normalized button press responses were binned across 10 s. Subsequently, covariance values were calculated between pairs of time series obtained for the same video clip—task combination (9 time series per combination). Covariance analysis compares the time courses of button press responses. If a pair of response patterns was similar throughout the video clip, the assigned covariance value was high. Conversely, if the response time courses differed, covariance was close to zero. This resulted in 36 values per video clip—task combination and, since each task was matched with 8 video clips, a total of 288 covariance values for each task. To control for video clip effects, across-task covariance was assessed. Here, covariance between time series obtained for the same video clip but for different tasks was calculated. The across-task covariance depended on gesture patterns in each video clip. Therefore, the comparison between same-task and across-task covariance revealed task-effects. Across-task comparisons resulted in 81 values per video clip and, since 12 video clips were presented, a total of 972 covariance values. The resulting 1836 covariance values (288 for task CON, ANY and REL, and 972 values for across tasks) were submitted to a 1-factor ANOVA with the 4-level factor “task comparison.” Subsequently, *post-hoc t*-tests were applied. Significance level was set at *p* < 0.05.

To control for global task performance, the binary responses to the control task were evaluated for each video clip—task combination. Percentage agreements were computed and averaged. A 2 by 2 ANOVA discerned differences between experiments (behavior, fMRI) and the groups with reciprocal experiment-video combinations (Group 1, Group 2).

### fMRI data acquisition and analyses

During fMRI, stimuli were presented with MR-compatible headphones and on a screen viewed via an angled mirror. Presentation and recording of button-press answers to control questions was conducted with Presentation software.

MR imaging was conducted on a 3 Tesla MR Scanner (Magnetom Prisma Fit, Siemens Medical Systems, Erlangen, Germany) at the Department of Psychiatry, Psychotherapy, and Psychosomatics of the RWTH Aachen University (Germany). Echo planar imaging (EPI) collected functional images sensitive to the blood oxygenation level dependent (BOLD) contrast (ascending interleaved acquisition of 33 slices; repetition time [TR] = 2,000 ms; echo time [TE] = 29.0 ms; flip angle [FA] = 77°; slice thickness = 3 mm; gap = 0.8 mm; field of view [FOV] = 192 mm; voxel size = 3 × 3 × 3 mm^3^). Slices were positioned oblique-transversally to achieve maximal brain coverage. Session length varied depending on the balanced video clip combination (average 891 volumes). Head movement was minimized using foam wedges to support the head position in a 20-channel head coil.

Functional MRI data analysis was conducted using the software Statistical Parametric Mapping (SPM8, http://www.fil.ion.ucl.ac.uk/spm/, implemented in MATLAB, version 8.2 R2013b, Mathworks, Natick, MA, USA). The first 4 volumes were discarded to remove the influence of T1 saturation effects. Images were spatially realigned to the mean image, normalized to the stereotaxic anatomical MNI space (Montreal Neurological Institute) with 2 mm isotropic voxels, spatially smoothed with a Gaussian kernel (isotropic 8 mm full width at half maximum, FWHM), and high-pass filtered at 0.008 Hz.

The volumes obtained during the presentation of video clips were analyzed with inter-subject covariance (ISC) mapping. Each video clip yielded an average of 275 volumes starting 6 s after the beginning of a narration. ISC values were calculated between pairs of time series obtained for the same video clip-task combination (9 time series for each combination). ISC analysis compares the time courses of neural responses. If, in a given voxel, a brain region showed similar response patterns throughout the video clip, the assigned covariance value was high. Conversely, if the activation time courses differed, covariance was low. Analogous to the behavioral experiment, this resulted in 36 values for each video clip-task combination and, since each task was matched with 8 video clips, a total of 288 covariance values for each task. Across-task covariance controlled for video-clip effects by calculating covariance between time series obtained for the same video clip but watched with across-task comparisons resulted in 81 values per video clip and, since 12 video clips were presented, a total of 972 across task covariance values in each voxel. In total (CON, ANY, REL, across task) 1,836 comparisons (ISC maps) were submitted to the analysis The covariance maps resulting from each data pair entered ANalysis of COVAriance (ANCOVA). The ANCOVA assessed task effects on regional covariance (4-level factor with the 3 tasks CON, ANY, REL, and the across-task comparisons). Gender and age differences (1 predictor each), group, as well as indicator variables for the video clips (with 10 predictors) were included in the model as predictors of no interest.

*F*-tests investigated effects between the 4 task levels. First, an ANOVA including the four predictors assessed overall task effects. Second, an *F*-test across task differences assessed task-specific effects for the CON task. *Post-hoc t*-tests assessed whether the conventionality task would increase local stimulus-induced BOLD fluctuation compared to the control tasks and the across-task condition. Correction for multiple testing was conducted according to random-field theory's family-wise error (FWE) correction at a significance threshold of p_FWE_ < 0.05. To further explore the task effects, the F-contrast was additionally displayed at an uncorrected threshold of *p* < 0.001 (Figure S1). Furthermore, exploratory t-maps contrasted the tasks with the across-task condition and with each other at an uncorrected threshold (*p* < 0.001; see **Figures 5**, **6**).

To investigate the localization within the language network, region-of-interest (ROI) analyses targeted the left IFG (Broca's area) and the left pSTG (Wernicke's area). The IFG ROI encompassed the anatomical regions IFG pars triangularis and IFG pars opercularis of the predefined AAL atlas (Tzourio-Mazoyer et al., 2002). The pSTG ROI included the posterior part of the AAL atlas region STG (MNI y-coordinate < −24). Across each ROI, averaged covariance values were extracted. Task effects were investigated with a 1-factor ANOVA with the 4 levels CON, ANY, REL, and across-task. Gender and age differences (1 predictor each), group, as well as indicator variables for the video clips (with 10 predictors) were included in the model as predictors of no interest. Statistics were performed with IBM SPSS (Statistics for Windows, Version 20.0, Armonk, NY: IBM Corp.). *T*-tests discerned the contribution of each factor level post-hoc (*p* < 0.05).

## Results

### Task-evoked behavioral responses

During the behavioral experiment participants watched 6 video clips of narrations (each about 9 min long) and pressed a button for each gesture they perceived as task-relevant. Participants gave 55.8 ± 62.1 (mean ± SD) responses per video clip. The CON task elicited the least amount of responses with on average 16.6 ± 22.2 button presses per video clip; the REL task elicited 41.1 ± 32.1 button presses; and the ANY task elicited most responses (109.6 ± 73.8).

**Figure 2:**
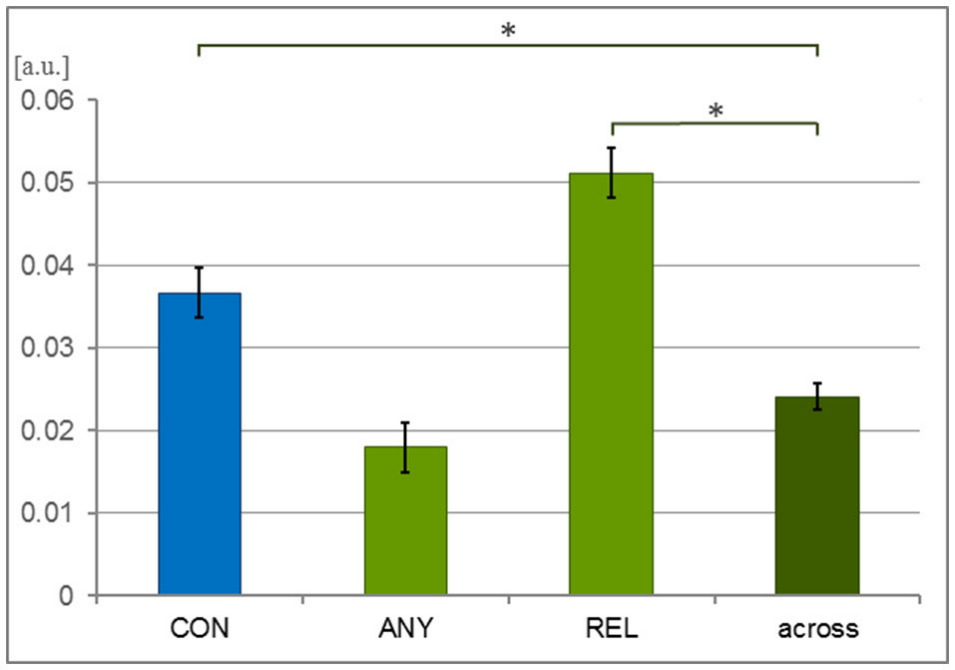
Covariance of behavioral responses. Thirty-six participants watched 6 video clips of 2 narrations each during a behavioral experiment. The time series of button press responses were analyzed with covariance analysis. To compensate for the event frequency, the response profiles were resampled to the same number of button presses in each condition. These response profiles were expected to be more similar—and thus their covariance higher—for the same task than across different tasks. Indeed, the CON task (conventionality) and the REL task (relation to speech) yielded higher covariance than the across-task comparison but not the ANY task (any hand movement). Bars represent mean ± SEM; ^*^p < 0.001; [a.u.], arbitrary unit.

Covariances were calculated between each time series obtained for the same videos. A 1-factor ANOVA confirmed a significant main effect for the 4-level factor “task comparison” [CON, REL, ANY, across-task; *F*_(3, 1, 832)_ = 27.50, *p* < 0.001; Figure 2]. As compared to across-task condition (0.024 ± 0.001), covariances were significantly higher within the CON task [mean ± SEM = 0.037 ± 0.003; *T*_(1, 258)_ = 3.63, *p* < 0.001] and the REL task [0.051 ± 0.003; T_(1, 258)_ = 7.72, *p* < 0.001] but only on a trend level within the ANY task [0.018 ± 0.003; *T*_(1, 258)_ = −1.82, *p* = 0.069]. Thus, tasks CON and REL yielded a specific response profile across participants.

During both the behavioral and the fMRI experiment participants decided after each video clip, which of the 2 narrations comprised more of the task-relevant gestures. Agreement among participants was 74.9 ± 15.4% during the behavioral experiment and 77.8 ± 13.5% during the fMRI experiment. The high agreement indicated that participants watched the video clips attentively and adhered to the tasks throughout both experiments. Furthermore, the finding indicates that participants interpreted the tasks in a comparable way. Half of the participants (Group 1) viewed the first half of the videos in the behavioral experiment and the other half during the fMRI experiment, whereas for the other half of the participants (Group 2) the allocation was switched. These groups differed on a trend level in the percentage of agreement rating [*F*_(1, 11)_ = 4.80, *p* = 0.051] but clearly neither a difference between behavioral and fMRI experiment [*F*_(1, 11)_ = 1.06, *p* = 0.326] nor an interaction emerged [*F*_(1, 11)_ = 1.10, *p* = 0.317]. This indicates similar task performance during both parts of the experiment and a moderate reliability.

### ISC mapping of fMRI data

**Figure 3:**
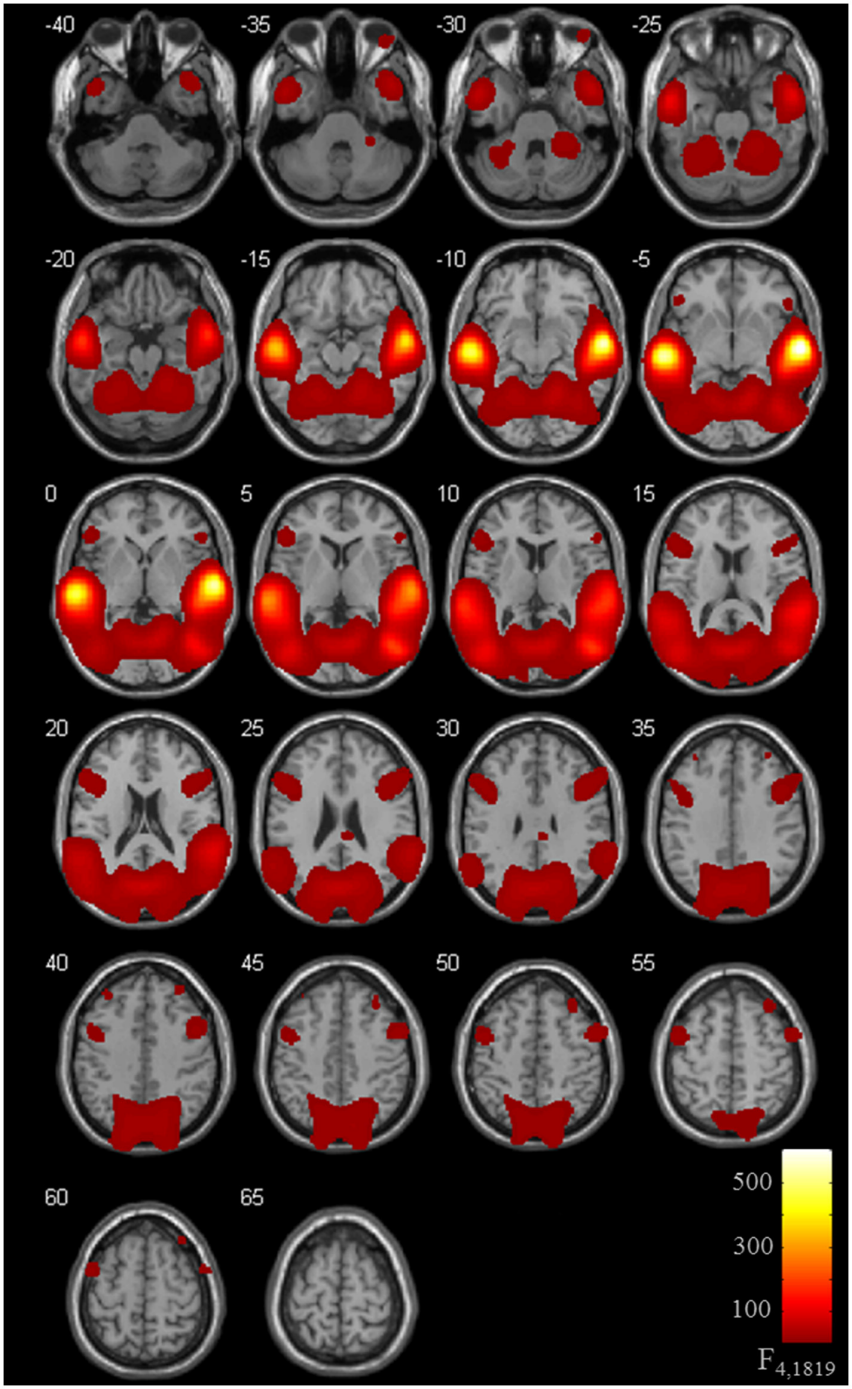
ISC for task effects. Thirty-six participants watched 6 video clips of 2 narrations each during fMRI scanning. Task effects on the inter-subject covariance (ISC) were modeled with predictors for the “CON,” “ANY,” and “REL” tasks compared to the across-task condition. The ANOVA including the intercept revealed an involvement of a distributed network with highest synchronous activity in auditory areas followed by the visual areas. Compared to previous ISC studies, the high involvement of frontal areas is remarkable (p_FWE_ < 0.05).

**Figure 4:**
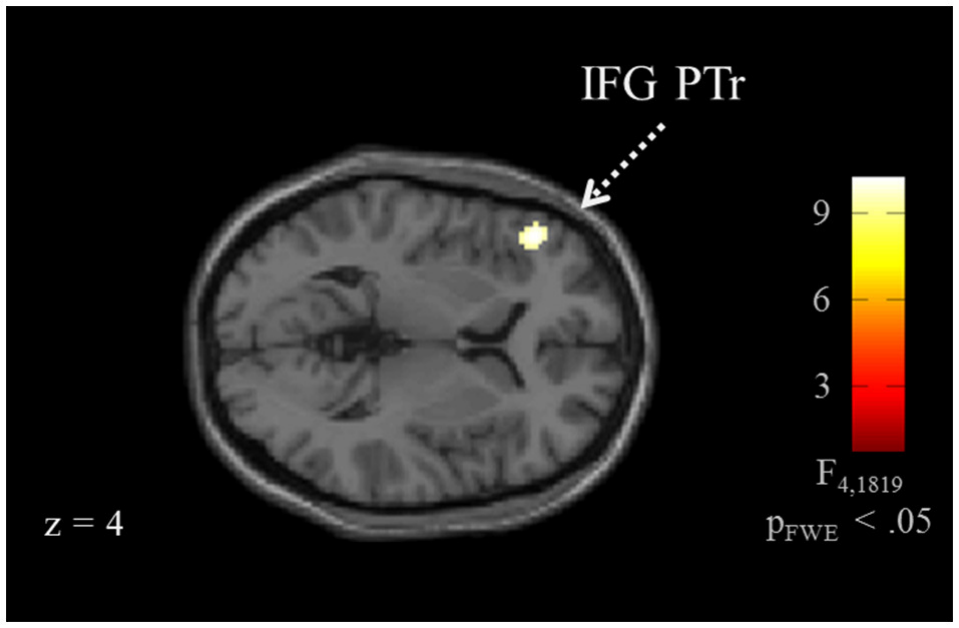
ISC for the conventionality task. The analysis for task effects, i.e., without the intercept, revealed a confined pattern at the left inferior frontal gyrus (IFG; p_FWE_ < 0.05) for the conventionality task (CON) over the control tasks and across task comparisons. For a visualization with the more liberal threshold uncorrected *p* < 0.001, see Figure S1.

**Figure 5:**
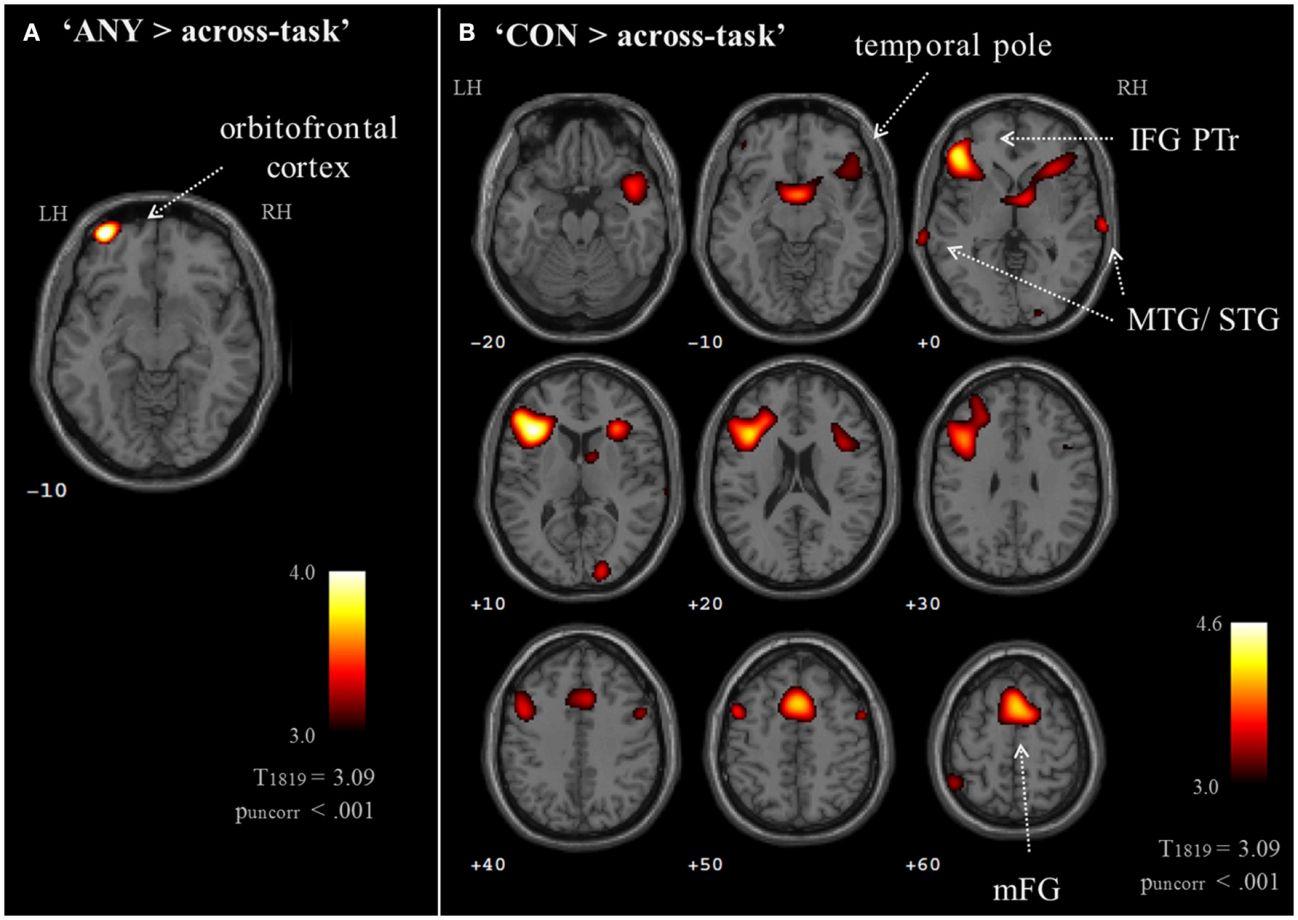
Exploratory whole brain maps for task effects. Exploratory t-maps (p_uncorr_ < 0.001; cluster threshold: 10 voxels) compared each task map to the across-task condition. **(A)** The contrast “ANY > across-task” revealed increased ISC at the left orbitofrontal cortex. **(B)** The contrast “CON > across-task” confirmed the left IFG response and further revealed contributions of the medial frontal gyrus, bilateral temporal cortices, right IFG, precentral gyrus, and cuneus. The contrast “REL > across-task” did not yield results on a whole-brain map. MTG, middle temporal gyrus; STG, superior temporal gyrus; IFG PTr, inferior frontal gyrus, pars triangularis.

**Figure 6:**
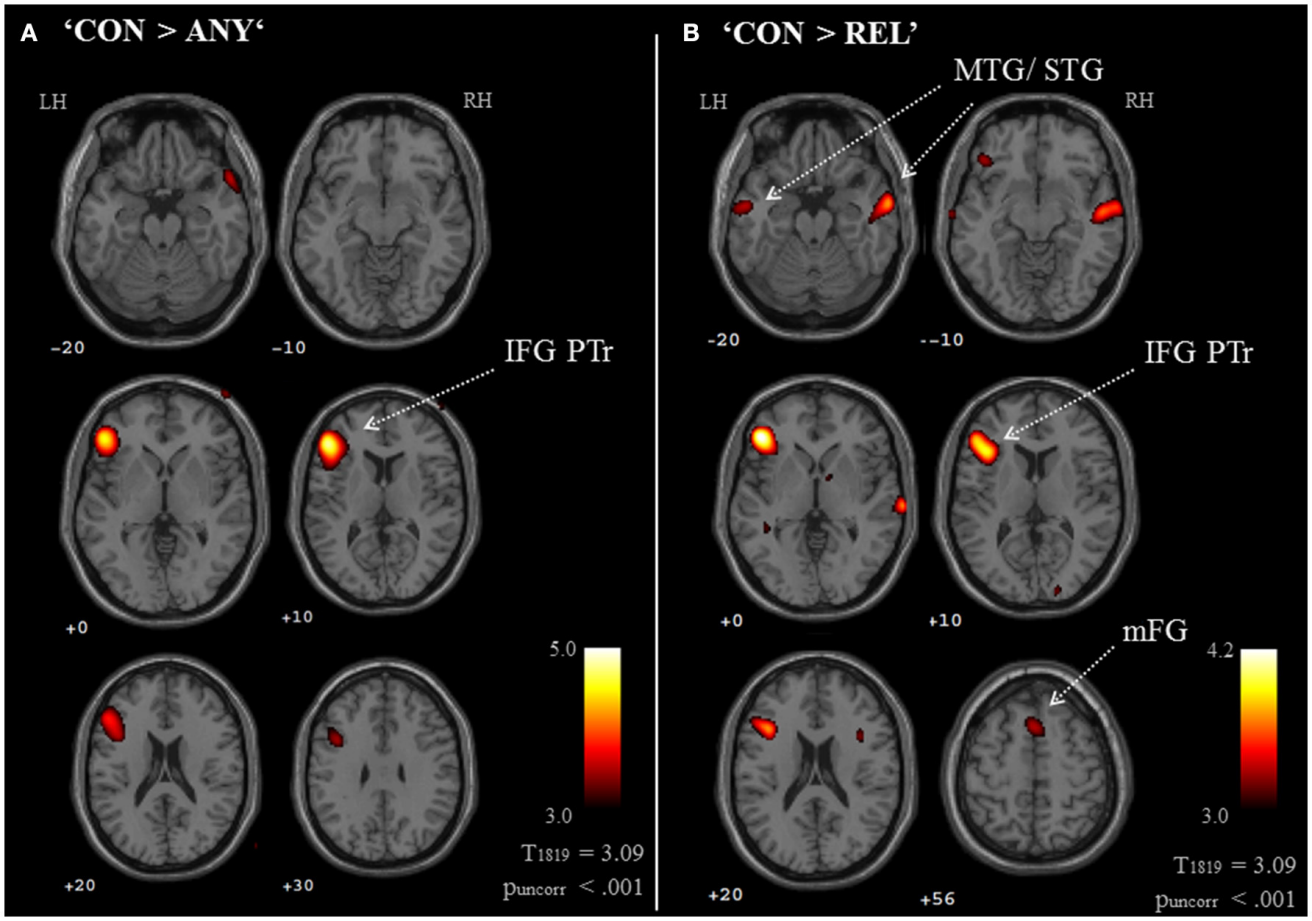
Exploratory whole brain maps for task-comparisons. Exploratory t-maps (p_uncorr_ < 0.001; cluster threshold: 10 voxels) compared each pair between the three tasks, which resulted in six contrast maps. Of those, only the two contrasts “CON > ANY” **(A)** and “CON > REL” **(B)** revealed clusters above threshold. Both maps confirmed the specific contribution of the IFG to the conventionality task (CON). Additionally, compared to the REL task (gesture-speech relation), the CON task increased covariance in the mFG, bilateral MTG, right IFG and right cuneus. IFG PTr, Inferior frontal gyrus, pars triangularis; mFG, medial frontal gyrus; MTG, middle temporal gyrus.

Brain activation of participants during the watching of multimodal narratives yielded significantly increased covariance in widespread networks (Figure 3). The involved regions covered most of the occipital, temporal, and parietal cortex as well as medial and ventro-lateral frontal cortex, with most robust statistics for the bilateral auditory cortex and adjacent superior and middle temporal regions (peak voxel MNI coordinates left: x = −62, y = −18, z = −6; right: x = 64, y = −10, z = −6). The F-test for specific effects for the CON task yielded a significant effect on synchronous neural activity in IFG pars triangularis (p_FWE_ < 0.05; Figure 4, Table 2). No other cluster survived at p_FWE_ < 0.05; in particular, no cluster could be observed in the pSTG, even after lowering the threshold to an exploratory *t* = 2.73 (voxelwise *p* < 0.001, see Figure S1, Table S1). Task specific effects in the IFG cluster were further supported by the exploratory t-contrasts directly comparing tasks. These contrasts were thresholded at an uncorrected *p* < 0.001. The cluster encompassing the IFG emerged not only for the contrast “CON > across-task” (Figure 5B, Table 3) but also for “CON > ANY” (Figure 6A, Table 3) and “CON > REL” (Figure 6B, Table 3). Furthermore, we did not observe such cluster for “ANY > across-task” (Figure 5A, Table 3) or “REL > across-task” (no suprathreshold clusters), demonstrating that the CON task specifically increased synchronous neural activity in IFG. As the exploratory contrasts “CON> across task” (Figure 5B, Table 3) and “CON > REL” (Figure 6B, Table 3) revealed, the conventionality task further recruited the medial frontal gyrus, bilateral MTG and the right cuneus.

**Table 2:** Cluster table for ISC maps.

**Peak voxel location**	**Cluster size (voxel)**	**Peak *F*-value**	**Peak voxel**			**Brodmann area**
			**x**	**y**	**z**	
***F*-TEST ACROSS TASKS (SEE FIGURE 3)**						
Right superior temporal gyrus	89,101	631.53	64	−10	−6	21
Right middle temporal gyrus		538.76	−62	−18	−6	21
Left middle temporal gyrus		197.81	50	−72	8	39
Left middle occipital gyrus		114.86	−46	−76	10	39
Left middle temporal gyrus		82.24	−46	−22	−16	21
Right calcarine gyrus		77.21	8	−72	10	30
Left middle temporal cortex		68.37	−46	−18	−18	21
Left middle temporal cortex		58.95	−42	−18	−12	21
Left middle temporal cortex		49.19	−40	−24	−8	21
Right middle temporal cortex		45.31	44	−18	−18	21
Right middle temporal cortex		43.30	42	−32	−12	21
Right superior occipital gyrus		29.01	18	−94	20	19
Left inferior temporal cortex		29.00	−42	−30	−14	36
Right hippocampus		28.75	38	−24	−10	–
Left middle occipital gyrus		25.16	−30	−76	0	18
Left middle temporal gyrus		22.77	−50	−34	−16	37
Right inferior frontal gyrus (pop)		22.31	44	16	26	9
Right middle temporal cortex		20.20	36	−26	−6	21
Left middle temporal cortex		19.86	−44	−42	−10	37
Right superior temporal cortex		19.43	36	−30	−4	21
Right precentral gyrus		18.34	56	4	50	6
Right inferior frontal gyrus (ptr)		12.40	54	30	0	47
Right inferior frontal gyrus (pop)		11.44	54	24	−8	47
Right middle frontal gyrus		10.59	32	44	40	9
Left superior parietal lobe		10.00	−30	−48	68	5
Right middle frontal gyrus		9.99	40	48	26	10
Right midcingulate cortex		9.88	6	−32	32	23
Left hippocampus		9.28	−38	−36	−10	–
Right angular gyrus		9.03	48	−60	50	40
Left inferior parietal lobe		8.91	−30	−48	40	7
Right posterior parietal cortex		7.87	6	−38	18	23
Left inferior frontal gyrus (ptr)	4,367	18.61	−48	20	22	46
Left precentral gyrus		16.50	−48	2	54	6
Left inferior frontal gyrus (ptr)		16.41	−52	30	6	45
Left middle frontal cortex	429	11.18	−34	42	42	9
Right anterior cingulate cortex	540	9.91	2	34	20	32
Left anterior cingulate cortex		9.82	2	38	14	32
***F*–TEST FOR THE CONVENTIONALITY TASK (SEE FIGURE 4)**						
Left inferior frontal gyrus (PTr)	45	9.86	−48	36	4	45

*Clusters with a minimum cluster size of 5 voxels and located within the brain mask are reported. T- or F-values are reported at p_FWE_ < 0.05; peak voxel coordinates are given in MNI-space. Pop, pars opercularis; PTr, pars triangularis*.

**Table 3:** Cluster table for exploratory ISC maps.

**Peak voxel location**	**Cluster size (voxel)**	**Peak *T*-value**	**Peak voxel**			**Brodmann area**
			**x**	**y**	**z**	
**ANY > ACROSS TASK (SEE FIGURE 5A)**						
Left middle orbital gyrus	194	4.15	−34	60	−10	11
**CON > ACROSS TASK (SEE FIGURE 5B)**						
Left inferior frontal gyrus (PTr)	3,817	4.73	−36	24	12	45
		4.67	−44	32	6	45
		4.03	−40	20	24	45
Right posterior medial frontal gyrus	2,431	4.35	2	14	56	8
Right inferior frontal gyrus (PTr)	1,746	3.86	32	26	10	45
Right temporal pole		3.71	44	10	−22	38
Right inferior frontal gyrus (PTr)		3.45	40	14	20	45
Right olfactory cortex		3.62	4	8	−10	25
Right putamen		3.22	22	20	−2	–
Right cuneus	215	3.63	18	−92	8	18
Right precentral gyrus	136	3.55	54	6	46	8
Right middle temporal gyrus	43	3.40	72	−24	0	22
Left inferior parietal lobe	22	3.33	−4	10	−12	25
Left middle temporal gyrus	26	3.32	−72	−30	−2	21
Left inferior parietal lobe	22	3.33	−4	10	−12	25
Left middle temporal gyrus	26	3.32	−72	−30	−2	21
Right caudate nucleus	10	3.25	10	4	10	–
**CON > ANY (SEE FIGURE 6A)**						
Left inferior frontal gyrus (PTr)	1,930	4.85	−48	36	6	45
		3.45	−42	16	24	45
Right temporal pole	10	3.34	54	20	−20	38
**CON > REL (SEE FIGURE 6B)**						
Left inferior frontal gyrus (ptr)	1,557	4.29	−46	36	2	45
		3.95	−40	22	12	13
		3.59	−40	20	24	45
Right middle temporal gyrus	624	3.86	64	−4	−16	21
Left middle temporal gyrus	184	3.40	−54	−6	−18	21
Left posterior medial frontal cortex	95	3.29	0	18	56	6
Right inferior frontal gyrus (PTr)	45	3.28	38	14	24	45
Right cuneus	13	3.15	20	−94	8	18

*Clusters with a minimum cluster size of 5 voxels and located within the brain mask are reported. T- values are reported at an uncorrected threshold of p < 0.001; peak voxel coordinates are given in MNI-space. PTr, pars triangularis*.

**Figure 7:**
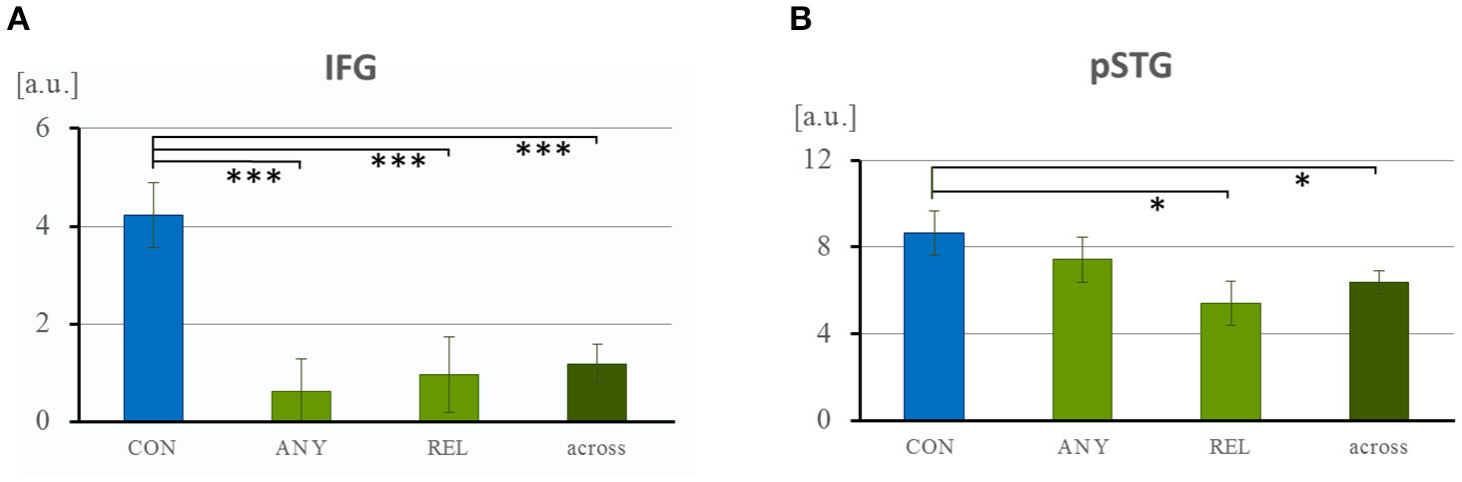
Region-of-interest analysis of the left IFG and pSTG. **(A)** Task-specific recruitment of the language networks left IFG and pSTG were examined with anatomically-defined ROIs. Across each ROI, averaged covariance values were extracted. **(A)** In the left IFG, neural synchrony was specifically higher during the CON task and close to zero for the other tasks and across tasks. **(B)** In the pSTG, the task effect was much weaker but ISC in general much higher. [a.u.], arbitrary unit; CON, task to attend to conventionality in co-speech gesture; ANY, task to attend to any hand movement; REL, task to attend to the gesture-speech relation. ^*^p < 0.05; ^***^p < 0.001.

The hypothesis-driven ROIs were selected from anatomical templates of left IFG and left pSTG. ROI analyses confirmed a selective response of the IFG. The ANOVA of the extracted ISC values yielded a significant effect of “task comparison” in the IFG [*F*_(16, 1, 797)_ = 5.84, *p* = 0.001] and, on a trend level, in the pSTG [*F*_(16, 1, 797)_ = 2.25, *p* = 0.081, n.s.]. Further, for the IFG ROI, *post-hoc t*-tests demonstrated increased values for the CON task (4.24 ± 0.66) in comparison to the ANY task [0.62 ± 0.68, *t*_(574)_ = 3.82, *p* < 0.001], to the REL task [0.10 ± 0.77, *t*_(574)_ = 3.21, *p* = 0.001], and to the across-task condition [1.19 ± 0.40, *t*_(1, 258)_ = 3.70, *p* < 0.001; Figure 7A]. In the pSTG, *post-hoc t*-tests revealed a significantly higher value for the CON task (8.65 ± 1.00) in comparison to the REL task [5.40 ± 1.02, *t*_(574)_ = 2.28, *p* = 0.023; Figure 7B] and to the across-task condition [6.36 ± 0.53, *t*_(1, 258)_ = 2.07, *p* = 0.039]. All other comparisons failed significance (all *p* > 0.16). Thus, direct hypothesis testing in the ROI analysis confirmed the role of the IFG in the conventionality encoding of co-speech gestures and further indicated the same preference for pSTG.

In summary, the conventionality tasks increased synchronization of behavioral responses, i.e., button presses mark co-speech gestures perceived as conventional. In addition, the CON task increased functional synchronization in the left IFG and, at a lower threshold, in the left pSTG. These cortex areas were not affect by the control tasks (task REL and task ANY).

## Discussion

Task instructions to detect conventionality in co-speech gestures increased synchronous activity in frontal language areas. Watching spontaneously produced narrations, the participants marked (behavioral experiment) or attended to (fMRI experiment) conventional aspects and to 2 control attributes of the observed gestures: the occurrence of any hand movement and the gesture-speech relation. Inter-subject synchronization during watching the multimodal narrations yielded patterns typically found for naturalistic stimuli. The conventionality task led to increased behavioral synchronization and thus aligned the perception of conventional co-speech gestures across participants. In the same vein, synchrony of neural activation increased in left IFG (Broca's area) and, on a trend level only, in pSTG (Wernicke's area) during the conventionality task over the detection of hand movements or gesture-speech relations. No other network was modulated by the detection of conventionality in co-speech gestures. Conceivably, the left IFG can be considered a core region for the processing of perceived conventionality in co-speech gestures during communication. In the following, we discuss the task-evoked attention to conventional aspects in co-speech gestures on the basis of the behavioral data. Then, we briefly consider the results regarding the ISC map across all tasks before turning to the task-based recruitment of the language networks. In particular, the left IFG and pSTG are connected to conventionality processing. Finally, we discuss possible implications of our results for the gestural origin of language hypothesis and for empirical research on Peirce's Universal Categories.

### Top-down task modulation and conventional co-speech gestures

For the detection of conventional aspects in co-speech gestures, no empirically-tested theory was available. Therefore, we designed tasks to match the theoretical concept of conventionality in co-speech gestures as inspired by Peirce's UCs and, in particular, the category of Thirdness. Our data confirm that task-induced modulation of neural processes during naturalistic stimulation may reflect complex cognition. In the behavioral experiment, the task had an effect on the covariance of response time series. In particular, indications of conventionality covaried across subjects more than with other tasks. Thus, participants agreed in parts on the occurrence of conventional gestures, indicating task reliability. Conceivably, the interpretation of gestural signs was reflected in the neural signature during the fMRI recording.

Participants decided similarly which of the two narrations comprised more of the task-relevant gestures. This reliability measure indicates that the participants understood the task in a comparable way. Further, the similarity of agreement scores between behavioral and fMRI experiment suggests that the task-according perception of gestures was similar; even in the absence of the behavioral on-line monitoring.

Behavioral covariance during the conventionality and the REL (relation between speech and gesture) tasks was significantly higher than the across-task covariance. The ANY task (any hand movement) did not increase covariance of response patterns as compared to across-task covariance. Possibly, the phrasing of this task was too vague to account for the complexity of gestural movements. In particular, co-speech gestures comprised not only strokes, but also other gesture phases such as the preparation phase, retraction phase, and various holds, i.e., pre-stroke and post-stroke holds (Kita et al., 1998; Kendon, 2004). Furthermore, several strokes or repetitions may enter into one gesture unit. As a potential source for additional variance prominently in the ANY condition, the participants' responses differed in marking strokes only, or other gesture phases as well. In conclusion, conventionality and communicative functions of co-speech gestures may be detected reliably even by untrained raters.

### ISC during natural communication

Natural communication is multimodal, encompassing not only spoken language but also head and shoulder movements, eye gaze, facial expressions, as well as manual gestures (e.g., Kendon, 2004). The communicated message is further embedded in the immediate context being built up during a longer dialogue or narrative, and in the broader context of background knowledge and internal states and processes (Small and Nusbaum, 2004). These factors are continuously integrated into an overall percept. Such interactions are not captured by more standardized experimental stimuli such as isolated vowels (e.g., Mathiak et al., 2000), words (e.g., Mathiak et al., 2002a) or sentences (e.g., Xu et al., 2005). In recent years, naturalistic stimulus materials have been utilized such as movie excerpts, video recordings of story narrations, and video games. Naturalistic stimuli comprise dynamic, contextual, and multimodal information during communication rendering them ideal for language studies (Tikka and Kaipainen, 2014; Willems, 2015).

ISC is independent from modeled responses and therefore is well suited for naturalistic stimulus material such as films under free viewing condition (Wilson et al., 2008; Bhavsar et al., 2014). Our study revealed high ISC across large parts of the cerebral cortex. ISC analysis yielded the highest statistics at bilateral superior and middle temporal areas followed by occipital and parietal regions. In general, this observation is consistent with previous reports for ISC during viewing of video clips and movies (Hasson, 2004; Bhavsar et al., 2014; Salmi et al., 2014). Indeed, the clusters encompassed all cortical regions frequently reported in fMRI studies investigating co-speech gestures in multimodal settings (for a review, see Yang et al., 2015). Conceivably, the ISC approach is sensitive to neural processing of communication signals encompassing co-speech gesture.

Similar to the other ISC study using video clips of multimodal communication (Wilson et al., 2008), superior and middle temporal areas were remarkably strong involved yielding higher statistics than occipital areas. This finding is remarkable since subjects attended to the gestures and fMRI created a relevant background noise (Mathiak et al., 2002b). Indeed, the auditory cortex is modulated by dynamic and congruent visual stimuli (Zvyagintsev et al., 2009) and vice-versa (Wolf et al., 2014). During face-to-face communication recruitment of auditory cortex is not only increased by language sounds, but also cross-modally by visual input such as lip movement and facial expressions (Hertrich et al., 2007; Okada et al., 2013; Strelnikov et al., 2015). In the case of co-speech gestures, Hubbard and colleagues (Hubbard et al., 2009) showed that bilateral non-primary auditory cortex exhibited greater activity when speech was accompanied by beat gestures than when speech was presented alone. These reports portray the impact of visual input on auditory cortex during multimodal communication.

Left posterior superior and middle temporal areas have a more direct role in language comprehension and are involved in phonetic, lexical-semantic, and higher-level sentence and text processing (see meta-analysis by Vigneau et al., 2006). A large-scale lesion-data analysis corroborates the role of the middle temporal gyrus in comprehension at the basic word-level (Dronkers et al., 2004), rendering this area highly relevant for lexical retrieval. These functions supported text understanding in our experiment as well, thus, explaining the locally high ISC values across conditions.

### Language networks and co-speech gestures

Here we demonstrate for the first time that the task-evoked attention on conventional aspects in spontaneously produced co-speech gestures particularly recruits left IFG and pSTG. The conventionality task aligned functional activation across participants in left IFG and, to a lesser degree, in pSTG. The latter structure may exhibit a lesser specificity to conventional gesture observation because it is tightly linked with auditory processing; in general, the pSTG is structurally and functionally associated with spoken language processing (Price, 2000).

### Left IFG in gesture processing

Left IFG and pSTG increase activation when a communication situation is presented (Courtin et al., 2011). The IFG has been ascribed a variety of communicative functions spanning multiple modalities, among those verbal inflection, syntactic, phonological, and semantic-lexical processing, language production, priming (communication-related) motor responses, recognition of meaningful hand actions, as well as processing of co-speech gestures, sign language, and emblems (Fadiga and Craighero, 2006; Grodzinsky and Santi, 2008). Since the recognition of its vital role in action-related cognition, the left IFG's main role is seen as a modality-independent semantic node “that supports symbolic communication” (Xu et al., 2009). In general, the left IFG has been suggested to support the finding of meaningful patterns and sequential information (Fadiga and Craighero, 2006; Tsapkini et al., 2008). Furthermore, it plays a role in integrating the meaning of words with other information such as gestures (co-speech gestures, lip-movements, facial expressions, body posture) and world knowledge (Özyürek, 2014). Indeed, its involvement in metaphoric gesture processing has directly been tested with transcranial direct current stimulation (Schülke and Straube, 2017). Our finding that IFG is specifically modulated during the detecting of conventional aspects in co-speech gestures further corroborates these views. As the ROI analysis demonstrated, covariance of functional responses in left IFG was significantly higher for task CON as compared to the control tasks (ANY, REL, across-task). Further, the tasks ANY and REL did not increase covariance as compared to the across-task comparison. This corroborates the model that the IFG supports semiotic rather than pure perceptual processes such as gesture detection and multisensory integration.

Several studies investigating neural correlates of co-speech gestures have reported a strong involvement of the left IFG in processing gesture—speech integration (as reflected in our REL task; e.g., (Willems et al., 2007, 2009; Straube et al., 2009, 2011; Schülke and Straube, 2017). However, both the whole-brain maps and the ROI analysis confirmed a particularly high sensitivity to the CON task. Since the REL task did not increase neural covariance as compared to the across-task condition, gesture-speech integration may constitute such a fundamental process during co-speech gesture perception that it remains unaffected during top-down elicited changes. Therefore, our task-based analysis may fail the investigation of this process.

In a small number of studies, no activation emerged in IFG during the viewing of video clips with co-speech gestures (Holle et al., 2008; Hubbard et al., 2009); in others activation increased in IFG for speech-gesture mismatches (Willems and Hagoort, 2007) or for self-adapters vs. iconic co-speech gestures (Green et al., 2009). While the former may be explained by the utilized tasks or the alteration of stimuli (such as editing, covered/blurred face or body), the latter were interpreted as an increase in integration load for gestures with no obvious relation to the accompanying speech. Raised cognitive demands due to task difficulty may be an alternative explanation for our results regarding the conventionality task. The focus on conventionality in co-speech gestures may have been more cognitively straining than the focus on hand movement and gesture-speech relation. While we cannot exclude this possibility, we rather propose that co-speech gestures produced in naturalistic, context embedded narratives particularly elicit semiotic processing and that this processing involves IFG. When co-speech gestures are presented in intentionally constructed mismatch situations or in unfamiliar combinations, the induced processes in the observer may be altered and the findings may not be transferable to real-world experiences (as has been discussed by e.g., Small and Nusbaum, 2004; Zaki and Ochsner, 2009). Indeed, even the experimental setup of presenting an emblematic gesture in combination with visual context or in isolation is sufficient to alter neuronal recruitment patterns (Villarreal et al., 2012). The reduced context may artificially enhance the observers' attention to the hand movements and may influence the observers' interpretation process (Andric and Small, 2012). By presenting the complete unedited narration, observers gained context information, and were flexible in their interpretation, i.e., which gestures they interpreted as being relevant. Thereby the naturalistic stimuli enabled a better approximation of perceptual processes during natural communication.

### Left pSTG in gesture processing

The posterior temporal region serves a rather wide range of functions. Its' involvement has been reported in experiments involving acoustic, visual, and motor stimuli as well as stimuli with social and interactive aspects (for review, see Hein and Knight, 2008). A meta-analysis found consistent involvement of left STG or STS areas in 31 imaging studies investigating co-speech gestures (Yang et al., 2015). However, emblems, as conventionalized gestures, yielded higher recruitment of the posterior temporal cortex than other co-speech gestures. Therefore, the pSTG was considered to contribute to the lexical retrieval for gestures—alike as for spoken words (Xu et al., 2009).

Based on the literature, we hypothesized involvement of the pSTG during semiotic interpretation of co-speech gestures and increased neural synchrony during conventionality detection (e.g., Xu et al., 2009). Further, the pSTG is known to contribute to multimodal integration during speech perception (see review in Campanella and Belin, 2007) and attention allocation even in the visual domain (Shapiro et al., 2002). Interestingly, no cluster survived after correction for multiple comparisons in the whole-brain ISC contrast. However, the subsequent ROI analysis revealed strong ISC throughout the condition with a trend-level effect of the four-level factor and significant increase in covariance for perceived conventionality (CON task) as compared to the REL task and the across-task condition. Since the overall engagement of pSTG during the viewing of co-speech gesture simultaneously to the listening to the speech was very high, the activations may have reached a ceiling and were modulated only to a small degree by the top-down task. In a similar vein, the processing of clear speech at the IFG but not at the pSTG benefitted from top-down information when listening to well intelligible speech (Davis et al., 2011). Most conceivable in our study, the information processed at the pSTG is represented fully and may not be further disambiguated by the top-down task (Frith and Dolan, 1997).

There are still controversies concerning the contributions of the pSTG to the processing of semantic information in gesture-speech integration. Willems et al. (2009) reported a sensitivity to the congruency of hand movement with speech in pantomimic but not in co-speech gestures. Similarly, Dick et al. (2009) compared self-adapter with iconic or metaphoric gestures and found a sensitivity of pSTS to hand movements, but not to their semantic message. On the other hand, the integration of hand movement with speech recruited pSTG for iconic and metaphoric gestures (Straube et al., 2010), for abstract more than for concrete gestures and for space-related more than for shape-related gestures (Nagels et al., 2013).

In general, networks encompassing the pSTG seems to support the multimodal integration and the interpretation of gestures accompanying speech (Hocking and Price, 2008; Dick et al., 2014; Özyürek, 2014). Our data in pSTG synchronization support this wide range of functions including a certain contribution to the semantic interpretation of co-speech gestures.

### Additional functions of the left IFG-pSTG network

Involvement of IFG and pSTG during the CON task may be driven by the processing of multimodal grammatical prosody, i.e., the syntactic structuring of an utterance by intonation changes (Guellai et al., 2014). In a meta-analysis on the language network's involvement in different linguistic tasks, both structures were relevant for grammatical processing (e.g., lexical categorization, syntax generation, or grammatical error detection; Vigneau et al., 2006). Co-speech gestures are produced in alignment with the concurrent speech and efficiently highlight relevant information in a similar manner as vocal intonation (for a review, see Krivokapi, 2014). In particular, beat gestures and discourse indices function as prosodic markers by highlighting particular words in a sentence and, therefore, indicate syntactic organization (Leonard and Cummins, 2011; Fricke, 2013; Mittelberg and Evola, 2014; Biau et al., 2016). Speech-congruent beat gestures yielded higher responses of the left IFG and middle temporal cortex as compared to incongruent gesturing (Hubbard et al., 2009; Biau et al., 2016) and compared to congruent movement of an object (Biau et al., 2016). Further, gesture mismatches elicited higher pSTG responses when produced within a sentence context as compared to a single-word context, confirming the relevance of co-speech gestures to narrative structures (Dick et al., 2009). In addition to rhythmic elements of beat gestures, image schemas represented in gestures may conceptualize the narrative structure of an utterance (Kimmel, 2013; Mittelberg, 2013a). The movement patterns of both beat gestures and of image schematic gestural representations carry conventional aspects (Cienki, 2010; Mittelberg, 2010, 2013b). However, empirical studies are lacking on the relevance of conventional aspects in multimodal grammatical prosody. In summary, the higher intersubject synchronicity in the left IFG-pSTG networks may be due to co-speech gestures which help to structure the spoken narrative.

### The gestural origin of language hypothesis

Our findings contribute to the debate about language evolution claiming that the origin of language does not lie in (involuntary) vocal exclamations but in voluntary gestural behavior (“gestural origin of language hypothesis”; Hewes, 1973; Corballis, 2010). In this light, hand movements may be regarded as potentially symbolic patterns containing sequential information in a similar manner as language. Comparable to language understanding, the concepts and actions expressed in gesture are understood by linking them to real-world knowledge (Fadiga and Craighero, 2006; Tsapkini et al., 2008). The left IFG (commonly labeled Broca's area) is a likely neural substrate for interpreting the symbols and habitual patterns (Rizzolatti and Arbib, 1998; Buccino et al., 2004). On an evolutionary scale, the observation of manual gestures may have shaped the lexico-semantic language areas (Courtin et al., 2011). The structural analogy for conventionality detection in verbal and gestural communication—as observed in our study—supports the idea that gestures served as a test bed for conventionality processing. Thus, conventional gesture comprehension promoted the development of higher functions involved in verbal communication.

### The universal categories as a tool for cognitive-semiotic investigations

We characterized and investigated conventional aspects in co-speech gestures by drawing on Peirce's Universal Categories. Peirce's semiotic triad is a central concept in the fields of linguistics, semiotics, and communication (Chandler, 2007). Conventionality is rooted in the category Thirdness, which was the focus of this study and fulfills a key function in the use and understanding of conventionalized communicative signs. As spoken and written words, emblematic gestures and manual signs in sign languages convey conventional lexical meanings as well as grammatical and pragmatic functions their understanding is dominated by Thirdness. By contrast, co-speech gestures may exhibit different kinds and degrees of conventionality (Mittelberg, 2006). Co-speech gestures typically receive their specific, local meaning in relation to other signs, such as speech and facial expressions, in a given context (“Secondness”). They may also only show the potentiality to express meaning or several different meanings (“Firstness”). However, in addition, conventional aspects of co-speech gestures may come to the fore based on abstract concepts and conceptual structures, e.g., conventional metaphors (Cienki and Müller, 2008; Cienki, 2010; Mittelberg, 2010, 2013a) action routines (Bressem and Müller, 2014; Ladewig, 2014), and/or pragmatic functions (Payrati and Teßendorf, 2014). Hence, co-speech gestures tend to be multifunctional and represent the full spectrum of Peirce's Universal Categories.

As pointed out earlier, symbolic and conventional meaning correspond to Thirdness in Peirce's UCs (Peirce, 1960; Potter, 1967). In neurocognitive investigations they are not only considered for gesture comprehension (Villarreal et al., 2012; Andric et al., 2013), but also for the perception of communicative signs in a broader sense (Donohue et al., 2005; Sato et al., 2009; Pulvermüller, 2013). For instance, pictures of objects activated the language network (like words) if they were perceived as symbolic (Tylén et al., 2009) and if they conveyed abstract social meaning (Tylén et al., 2016). Furthermore, Peirce's Universal Categories have inspired theories regarding the emergence of social conventions and symbolic communication during language evolution (Deacon, 1997) and during child development (Daddesio, 1994). These examples emphasize the applicability of Peirce's semiotic theory for the investigation of communicational signs and behaviors with respect to various sign properties. However, empirical studies are still scarce and ways of application vary across studies; thus, the picture is still incoherent and does not warrant generalizations (Fusaroli and Paolucci, 2011; Zlatev, 2012). With the present study we contribute to the endeavor of operationalizing Peirce's semiotic theory for empirical investigations within the fields of social cognition and neuroscience. Indeed, the successful implementation of a conventionality task may be considered a first step toward an empirical foundation of the Universal Categories.

All in all, modulating perception with tasks based on Peirce's cognitive-semiotic dimensions enabled the holistic neurosemiotic investigation of conventional aspects in co-speech gestures as they naturally occur during communication. Such a semiotic approach offers a novel means to investigate the neural representation of communication in naturalistic stimuli.

### Limitations

Although freely performed monologs offer superior ecological validity compared to more traditional experimental stimuli, they are less well controlled. The occurrence of speech and gesture is not balanced or randomized and the videos are inherently heterogeneous. We sought to control for this variability by balancing the narrations for length, gender of the speaker, topic, and number of produced strokes. Furthermore, we combined one video-clip with two tasks. Nevertheless, a full task permutation was unfeasible and, therefore, differences in language and movement complexity as well as physical characteristics may coincide partially with a measured construct.

The task instruction was designed to reflect the concept of conventionality in co-speech gestures according to Peirce's UCs. Peirce's pragmatist approach to communication processes is well suited for systematic analyses. However, this is the first attempt to empirically test task-directed conventionality perception and direct evidence for task effectiveness is lacking. Nevertheless, the participants' responses indicated an understanding of our concept of conventionality; the tasks produced reliable results which were comparable for both the behavioral and fMRI experiment and for both sets of video-clips.

ISC can reveal synchronization in brain activation across subjects but does not inform about the direction of activation changes, i.e., increase or decrease of activation after a specific stimulus. We chose this model-free approach with the experimental manipulation of gestures to explore the neural correlates of perceived conventionality in co-speech gestures independent of theoretical models. The behavioral responses revealed that participants responded to similar gestures as conventional suggesting that neural activations may be expected in preparation of the responses. The ISC analysis revealed that the modulations of left IFG and pSTG activations were stronger during conventionality processing.

## Conclusion

Simple instructions to laymen subjects enhanced the processing of conventional aspects in spontaneously produced co-speech gestures, as revealed by increased inter-subject synchronization of behavioral and neural responses. In the language network, functional synchronization was significantly increased in the left IFG (Broca's area) and, to a lesser degree, in the left pSTG (Wernicke's area). In line with studies on highly conventionalized manual signs such as emblems and sign language, conventional aspects of discourse-integrated gestures seem to be processed in the language networks. In general, the interpretation of conventional signs, based on either rules or habits, may rely on neural mechanisms subserving language comprehension and may even be a central building block of the human language facility.

## Ethics statement

This study was carried out in accordance with the Code of Ethics of the World Medical Association (Declaration of Helsinki, 2008). All subjects gave written informed consent in accordance with the Declaration of Helsinki. The protocol was approved by the local Ethics Committee (Uniklinik RWTH Aachen).

## Author contributions

DW: planned the study, collected the data, analyzed the data, and wrote the manuscript; L-MR: planned the study, assisted in data collection and edited the manuscript; IM: planned the study and edited the manuscript; MK: assisted in data collection and data analysis; KM: planned the study, assisted in data analysis and edited the manuscript.

### Conflict of interest statement

The authors declare that the research was conducted in the absence of any commercial or financial relationships that could be construed as a potential conflict of interest.
