# Supplementary material for: Perceived Conventionality in Co-speech Gestures Involves the Fronto-Temporal Language Network
Source: Front Hum Neurosci. 2017 Nov 30;11:573. doi: 10.3389/fnhum.2017.00573 (PMC5714878; doi:10.3389/fnhum.2017.00573)
Supplement: Supplementary file 1 [file DataSheet1.PDF]

Supplementary Figure and Table

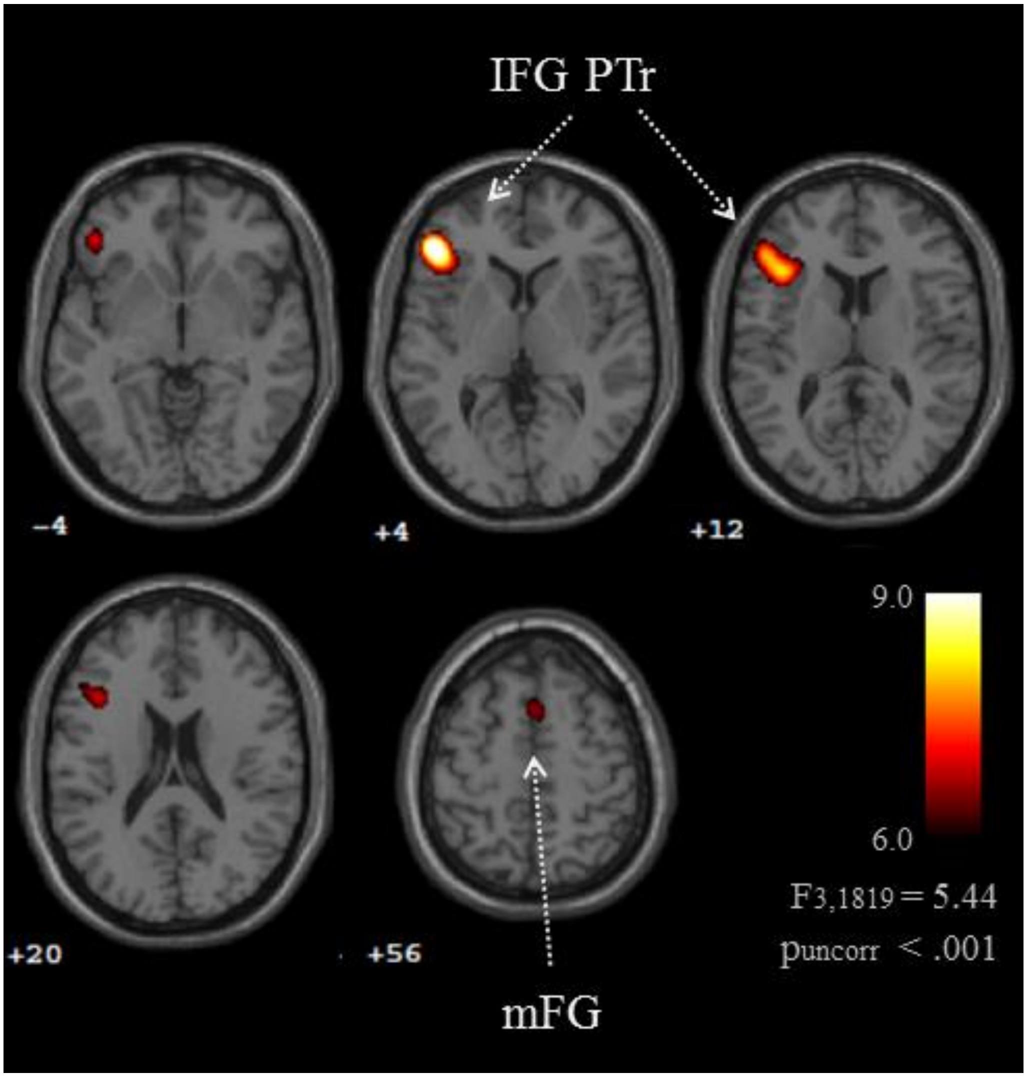

Figure S1: ISC for task CON

At a conservative threshold, the conventionality task led to higher synchronized activity in only at the left inferior frontal gyrus, pars triangularis (IFG PTr) compared to control and across task (see Fig. 3, $p_{FWE} < .05$). At a statistical threshold uncorrected for multiple testing across the volume ($p_{uncorr} < .001$; cluster threshold 10 voxels), two small additional clusters emerged at the medial frontal gyrus (mFG) and the left orbitofrontal cortex (not visible in the figure). Notably, no activation cluster emerged that indicated involvement of the temporal cortex.

Table S1: Cluster table for supplementary figures S1 and S2

peak voxel location	cluster size [voxel]	peak F-value	peak voxel x	y	z
**F-test for tasks** (p_{uncorr} < .001; see Fig. S1)					
left inferior frontal gyrus (PTr)	1361	9.86	-48	36	4
left inferior frontal gyrus (PTr)		6.35	-40	20	24
right posterior medial frontal cortex	296	6.57	2	14	56
left middle orbital gyrus	45	6.57	-36	60	-10

Clusters with a minimum cluster size of 10 voxels and located within the brain mask are reported. T- or F-values are reported at p_{uncorr} < .001; peak voxel coordinates are given in MNI-space. Abbreviations: PTr: pars triangularis.
